# Editorial: Accomplishments, Collaborative Projects and Future Initiatives in Breast Cancer Genetic Predisposition

**DOI:** 10.3389/fonc.2019.00841

**Published:** 2019-08-28

**Authors:** Paolo Peterlongo, Luis G. Carvajal-Carmona

**Affiliations:** ^1^Genome Diagnostics Program, IFOM The FIRC Institute for Molecular Oncology, Milan, Italy; ^2^Genome Center, University of California, Davis, Davis, CA, United States; ^3^Department of Biochemistry and Molecular Medicine, School of Medicine, University of California, Davis, Sacramento, CA, United States; ^4^Population Sciences and Health Disparities Program, University of California Davis Comprehensive Cancer Center, Sacramento, CA, United States

**Keywords:** breast cancer genetic predisposition, GWAS, VUS, PRS, splicing

Since the discovery of breast cancer genes *BRCA1* and *BRCA2 (BRCA1/2)* over two decades ago, much has been accomplished in the field of breast cancer genetic predisposition. On one hand, novel genes harboring rare pathogenic variants, most of which act in the same *BRCA1/2* pathway, causing increasing disease risk have been identified. In addition, several single nucleotide polymorphisms (SNPs) that modify the breast cancer risk in individuals with *BRCA1/2* mutations are now known. These moderate-to-high penetrant genetic variants now represent key elements for improving risk prediction in familial cases. On the other hand, hundreds of common low-risk SNPs have been discovered and can be incorporated into prediction models to improve the identification of women at risk of breast cancer in the general population. Moreover, multifactorial analyses, family studies and high throughput functional assays have been developed to validate candidate genes, classify the variants of uncertain significance (VUS) detected by gene-panel next generation sequencing in clinical and research settings, and to measure the risk magnitude conferred by known pathogenetic variants. Articles in the present Frontiers in Oncology e-book explore these aspects of breast cancer predisposition further.

Individuals who carry *BRCA1/2* pathogenic variants have an average cumulative risk of developing breast cancer, by age 80 years, of ~70% (1). Thanks to the efforts of the collaborators of the *PALB2* Interest Group (http://www.palb2.org/), *PALB2* is now considered the third high-risk gene with pathogenic variants associated with 44% lifetime risk of developing breast cancer (2). The moderate-penetrance genes *ATM* and *CHEK2* are also associated with breast cancer, conferring a 20% average lifetime risk (3, 4). More recently, *BARD1, RAD51D, BRIP1*, and *RAD51C* have been proposed as risk factors for triple-negative breast cancer [TNBC; (5)], indicating that the risk associated with pathogenic variants in each gene may vary by tumor subtype. Support for this hypothesis is the latest emerging breast cancer gene *FANCM* which has also shown to confer a higher risk for TNBC (6–8). All these genetic factors explain only about half of the familial cases, hence novel breast cancer genes or alleles are yet to be detected (9). In this e-book, the impact of *BRCA1/2* mutations and of novel genes was investigated in unexplored populations and in breast cancer progression. Solano et al. studied the *BRCA1/2* mutation spectra in high-risk Ashkenazi Jewish population from Argentina. They reported that, in addition to carriers of known Ashkenazi founder mutations, up to 7% of tested individuals were positive for other *BRCA1/2* pathogenic variants. In a second study, Torrezan et al. performed whole exome sequencing in Brazilian breast cancer probands that were negative for causal variants in most known predisposition genes. Beside a very rare and novel pathogenic variants in *ATM* and *BARD1*, respectively, the authors found rare and possibly damaging variants in several candidate genes. Tang et al. aimed at the identification of genes associated with the progression of breast cancer. The authors developed a free-scale gene co-expression networks to explore associations between gene sets and clinical features, and to identify candidate biomarkers. Breast cancer is not exclusively a female disease and about 1% of all cases arise in males. As reviewed by Rizzolo et al., 13% of male breast cancer (MBC) are due to pathogenic variants in *BRCA2* while *CHEK2* and *PALB2* account for a smaller proportion of cases. In their study these authors suggest that monoallelic mutation of *MUTYH* gene, which cause the recessive MUTYH-associated polyposis (MAP) syndrome may also cause MBC.

The identification of common SNPs associated with breast cancer risk and of those that modify the breast cancer risk in individuals with *BRCA1/2* pathogenic variants is the most significant success of the Breast Cancer Association Consortium (BCAC, http://bcac.ccge.medschl.cam.ac.uk/) and of the Consortium of Investigators of Modifiers of B*RC*A*1/2* (CIMBA, http://cimba.ccge.medschl.cam.ac.uk/). A meta-analysis of genome wide association studies (GWAS), which combined data from 122,977 breast cancer cases and 105,974 controls, resulted in the identification of a total of 172 risk-associated SNPs explaining about half of the familial relative risk [the risk of first-degree relatives of breast cancer patients of developing the disease; (10)]. Ten additional SNPs were found by the analyses of breast cancer cases with estrogen receptor (ER) negative tumors (11), bringing the total number of known breast cancer SNPs to 182 SNPs. While individually these risk factors are not clinically relevant, they can be combined into polygenic risk scores (PRS) that can be predictive of cancer risk in *BRCA1/2* mutation carriers and in the general population (1, 12). As discussed in this e-book, in a review by Rivandi et al., many of these GWAS-identified SNPs are located outside coding regions and are tags for mostly unknown, causal or functional variants. Hence, their identification would provide better estimates of the explained familial relative risk, thereby improving polygenetic PRSs and increase our understanding of the biological mechanisms involved in breast cancer susceptibility. The success of BCAC and CIMBA in identifying several low risk alleles, resides in the capability of coordinating the efforts of over 180 worldwide groups or studies contributing DNA samples and data from breast cancer cases and control and from *BRCA1/2* mutation carriers. The French Genetic Modifiers of *BRCA1* and *BRCA2* (GEMO) Group, described in this e-book by Lesueur et al., is one of the larger studies within CIMBA. GEMO was initiated in 2006 and today involves 32 clinics and 17 diagnostics laboratories that, as of April 2018, collected 5,303 participants.

As discussed above, many variants in *BRCA1/2* and in other established or candidate breast cancer genes have uncertain clinical significance. These VUSs, which are typically rare missense variants, represent a serious clinical problem as carrier risk estimates are often unclear. The Evidence-based Network for the Interpretation of Germline Mutant Alleles (ENIGMA; https://enigmaconsortium.org) was formed to determine the clinical significance of variants in *BRCA1/2* and other known or suspected breast cancer genes (13). To this aim, ENIGMA gathers pathologists, epidemiologists, geneticists, bio-informaticians, genetic counselors, and molecular biologists into working groups to assess the clinical relevance of variants by applying statistical approaches and multifactorial likelihood models, studying tumor markers, or performing functional assays. Two articles in this e-book provide insights into VUSs classification. The first study, by Zuntini et al., investigated whether co-segregation analyses, integrated with functional data and *in silico* predictions, could improve VUSs interpretation and counseling in carrier families. In the second study, Caleca et al., used an *in vitro* assay specifically designed to test the *BRCA2* and *PALB2* gene products interaction and showed initial pathogenicity evidence for two very rare missense variants in these genes. A special class of VUS are those suspected to cause mRNA splicing defects. One of the ENIGMA working groups was established to improve the clinical classification of likely spliceogenic variants. Members of this working group contributed articles exploring some of the aspects of this variant class. For example, Fraile-Bethencourt et al. identified eight spliceogenic variants in exon 16 of *BRCA2* by minigene assay, highlighting the efficiency of this approach for clinical classification. *In silico* tools for splicing defect prediction may play a key role in VUS analysis. Moles-Fernández et al. used 99 *in vitro*-validated variants to evaluate the performance of six commonly used splicing *in silico* tools. Finally, Farber-Katz et al. conducted analyses of *BRCA1/2* variants using a novel RNA-massively parallel sequencing assay capable to perform quantitative and qualitative analysis of transcripts. Similarly, Lattimore et al. utilized targeted RNA-seq to re-assess *BRCA1/2* mRNA isoform expression patterns in lymphoblastoid cell lines. Recommendations from these two studies will facilitate the application of targeted RNA-seq approaches for the quantitative characterization of *BRCA1* and *BRCA2* germline splicing alterations.

In summary, articles in the present e-book move the field of breast cancer genetics in several aspects, ranging from characterizing genetic variation in new populations to developing and applying tools for variant re-classification. Since the discovery of the role of *BRCA1/2* on breast cancer risk, much has been learned and through the tremendous international, multi- and inter-disciplinary efforts of consortia such as BCAC, CIMBA, and ENIGMA. The next decade promises to illuminate many new aspects of breast cancer risk prevention on families and in the general population.

## Author Contributions

PP and LC-C wrote this manuscript.

### Conflict of Interest Statement

The authors declare that the research was conducted in the absence of any commercial or financial relationships that could be construed as a potential conflict of interest.
